# Experiences of stigma, psychological distress, and facilitative coping among pregnant people with gestational diabetes mellitus

**DOI:** 10.1186/s12884-023-05949-z

**Published:** 2023-09-07

**Authors:** Shufang Sun, Jennifer Pellowski, Claire Pisani, Diksha Pandey, Mallory Go, MyDzung Chu, Jenny Ruan, Erika F. Werner

**Affiliations:** 1 Department of Behavioral and Social Sciences, Brown University School of Public Health, 121 S. Main St, Providence, RI 02903 USA; 2 International Health Institute, Brown University School of Public Health, Providence, USA; 3 Mindfulness Center, Brown University School of Public Health, Providence, USA; 4 The College at Brown University, Providence, USA; 5 Watson Institute for International and Public Affairs, Brown University, Providence, USA; 6 Tufts Clinical and Translational Science Institute, Boston, USA; 7 Department of Obstetrics and Gynecology, Tufts Medical Center, Boston, USA; 8 Tufts University School of Medicine, Boston, USA

**Keywords:** Gestational diabetes Mellitus, Stigma, Mental Health, Pregnancy

## Abstract

**Background:**

Gestational diabetes mellitus (GDM) has been rising in the United States, and it poses significant health risks to pregnant individuals and their infants. Prior research has shown that individuals with GDM also experience prevalent stress and mental health issues, which can further contribute to glucose regulation difficulties. Stigma associated with GDM may contribute to these mental health challenges, yet there is a lack of focused research on GDM-related stigma, its impact on psychological health, and effective coping mechanisms. Thus, this qualitative study aims to understand individuals’ experiences related to GDM stigma, mental health, and facilitative coping.

**Methods:**

In-depth, semi-structured interviews were conducted with 14 individuals with a current or recent (within the last year) diagnosis of GDM. Thematic analysis was employed to guide data analysis.

**Results:**

Four themes emerged from data analysis: (1) experience of distal GDM stigma including stigmatizing provider interactions, stigma from non-medical spaces, and intersecting stigma with weight, (2) internalized GDM stigma, such as shame, guilt, and self-blame, (3) psychological distress, which included experiences of stress and overwhelm, excessive worry and fear, and loneliness and isolation, and (4) facilitative coping mechanisms, which included diagnosis acceptance, internet-based GDM community, active participation in GDM management, social and familial support, and time for oneself.

**Conclusions:**

Findings demonstrate the relevance of GDM stigma in mental health among people with GDM and the need for addressing GDM stigma and psychological health in this population. Interventions that can reduce GDM stigma, improve psychological wellness, and enhance positive coping may facilitate successful GDM management and healthy birth outcomes. Future quantitative, theory-driven research is needed to understand the prevalence of GDM stigma experiences and mechanisms identified in the current study, as well as among marginalized populations (e.g., individuals of color, sexual and gender minorities).

Gestational diabetes mellitus (GDM), glucose intolerance during pregnancy, poses significant health and well-being challenges to affected people and their infants, including putting postpartum individuals at higher risk of developing Type 2 diabetes mellitus later in life [1]. In the United States, the prevalence rate of GDM has been rapidly increasing; the prevalence of GDM in 2006 was 4.6% compared to 8.2% in 2016 [2], with higher prevalence rates among some racial/ethnic groups, such as non-Hispanic Asian (14.7%) and American Indian/Alaska Native (11.8%) [3]. The economic burden of GDM is also high: in 2017, GDM was estimated to contribute $1.6 billion in medical costs [4]. In the US, GDM is often diagnosed between 24 and 28 weeks gestation through an initial and follow-up oral glucose tolerance test (OGTT) [5]. Management of GDM may take the form of lifestyle management or lifestyle plus medication management [6]. Daily glucose monitoring and additional growth ultrasounds are also standard of care [7]. Poorly controlled GDM is associated with various potential pregnancy, birth, and health complications for parents (e.g., higher risks for cesarean birth, preeclampsia, postpartum hemorrhage, and future Type 2 diabetes and cardiovascular disease) and infants (e.g., large for gestational age, stillbirth, metabolic issues in adulthood) [8–14].

Previous qualitative studies have found that following a GDM diagnosis, people with GDM experience high levels of stress, face major barriers to incorporating nutritional recommendations into their daily social lives, and possess an overall lack of education (e.g., on healthy dietary choices, skills necessary to make lifestyle changes and support glucose management) [15–17]. Further, people with GDM also experience prevalent mental health concerns, such as depression, anxiety, and stress [18, 19]. Attending to the psychological health of individuals with GDM remains critically important. One potential contributing factor to the well-being of people with GDM may be stigma. Broader literature on chronic conditions, such as cardiovascular disease, diabetes, and cancer, has linked experiences of stigma with decreased quality of life, psychosocial issues, and poor disease management [20, 21]. Despite these common associations, the sources and manifestations of stigma differ by chronic diseases based on their disease-specific features [21]. For example, the emerging research on diabetes outside of pregnancy (namely, Type 1 or Type 2) finds that experiences of stigma are associated with negative impacts on mental well-being, eating patterns, and healthcare engagement including glucose monitoring [22–25].

More recently there has been increased focus on investigating the experiences of stigma among people diagnosed with GDM. Davidsen and colleagues recently conducted a scoping review and found 44 articles that investigated experiences of individuals with GDM (e.g., facilitators and barriers of care, women’s health behaviors) to extract relevant findings on overt discrimination and internalized stigma (namely guilt and shame about their diagnoses) [26]. Although many of these articles discussed topics relevant to stigma, few specifically focused their investigation on stigma or employed a stigma lens [27]. While there has been some movement in the literature to investigate experiences of stigma associated with GDM, the literature is still lacking in terms of four key elements. First, although a recent qualitative study investigated experiences of GDM stigma among Danish women with GDM [27], less is known regarding women’s experience specifically in the U.S. despite its growing epidemic. Second, the present literature often lumps stigma into psychosocial challenges, and there is a lack of investigation on how stigma itself may impact psychological health and, more importantly, facilitative coping (i.e., strategies and processes through which individuals employ or undergo to support one’s psychosocial and behavioral health and adjustment to a new medical condition) to reduce stigma and improve well-being. Understanding the mechanisms of stigma and effective coping can be crucial to guide future intervention efforts. Third, while there is a more robust literature focused stigma associated with pre-existing diabetes, less is known about stigma and GDM, which by definition is a shorter-term complication but does lead to intensive use of the medical system. Fourth, there is a lack of qualitative research and analyses that are aimed at building theoretical frameworks on GDM-related stigma that can be applied and tested in future research.

The current study investigates pregnant or postpartum individuals’ experiences of stigma (e.g., internalized stigma and overt discrimination and prejudice) following their diagnosis with GDM. The aims of this study are to (1) investigate the experiences of stigma among individuals diagnosed with GDM in the United States and the impact of stigma on psychological health, (2) identify facilitative coping strategies used by these individuals to address stigma and improve well-being, and (3) build a conceptual model from the qualitative findings to inform future quantitative research and intervention development.

## Method

### Recruitment & participants

The study was approved by the Institutional Review Board (IRB) at Brown University. Information about the study was promoted at regional OB/GYN clinics affiliated with Women & Infants Hospital and Tufts Medical Center, as well as on relevant social media platforms (Facebook groups, Reddit community). Interested individuals were invited to read more information about the study (including its risks and benefits), provide consent for participation via clicking on commensurate button to continue, and complete a brief online screening survey to determine initial eligibility. To be included in the study participants needed to (a) be 18 years of age or older; (b) have had a pregnancy complicated by GDM either currently or within the past 12 months; (c) be able to read and speak English; (d) reside in the U.S.; (e) have access to a device that allows for an internet-based conference video interview; and (f) not have pre-pregnancy diabetes or GDM in a more distant pregnancy (i.e., more than a year ago). If determined to be preliminarily eligible, participants were then re-directed to fill out a contact form. A Research Assistant (RA) contacted potentially eligible participants and scheduled a Zoom-based interview with investigators.

A total of 14 individuals participated in the study. Ages ranged from 24 to 38 years old (*Median* = 32). Among them, 11 were pregnant at the time of the interview (average 31.3 weeks gestation), and three were postpartum (within one year). The majority of participants identified as non-Hispanic White (*n* = 12; 85.7%) and two identified as Asian (14.3%). Most participants (*n* = 9; 64.3%) were working full-time and four participants (28.6%) were unemployed/not working (7.1% or one participant did not answer). Table 1 provides a detailed summary of participant sociodemographic characteristics.

### Interview protocol

Two PhD-level psychologists (SS, JP) conducted individual interviews with participants. Before the interviews began, researchers explained confidentiality, data security, and consent with participants and ensured participant understanding prior to starting the interview. Interviews lasted 60–90 min in duration. Each participant received a $30 Amazon gift card as a compensation for their participation in the study. Interviews were conducted during July 2022 to November 2022. All interviews were recorded and professionally transcribed.

Following Strauss and Corbin’s [28] suggestions on a tunnel approach to the interview process, interviews started with rapport building, followed by questions related to GDM (e.g., experiences of diagnosis, awareness of any risk factors, etc.), lifestyle adjustment and current treatment/management, experience of psychological stress and distress, and coping (e.g., What strategies have supported your mental health and what have not?).

### Data analysis, research quality and trustworthiness

We employed thematic analysis to synthesize the data [29]. Specifically, this entails a six-step process. The first phase involved the research team, including two interviewers (SS & JP) and three RAs (CP, DP, & MG) familiarizing themselves with the data through repeated reading and transcription (RAs performed part of interview transcription for the purpose of data familiarity). Phase two involved generating codes across the entire dataset. Each transcript was coded by two paired RAs independently. Coding was data-driven, and RAs met regularly to discuss the coding process and resolve discrepancies. In phase three, investigators reviewed all codes relevant to stigma, their sources, and their impact on participants to uncover patterns and relationships in the dataset. Investigators re-read the transcripts to contextualize codes and combined codes into potential themes and subthemes. In phase four, all themes (main themes and subthemes) were reviewed. Investigators discussed the relationship among the themes, presented them in a more systematic way as a map/figure, and refined themes (e.g., merging subthemes that had overlapping meanings). Phase five involved defining and naming each theme to ensure that each one reveals a distinct and unique aspect of the data. Phase six involved writing and preparing the manuscript, including selecting quotes that represent the essence of each theme. Pseudonyms for participants are used in the presentation of the results.

We used multiple strategies to ensure the quality and trustworthiness of the study [30, 31]. Credibility was ensured through prolonged engagement with the data, including sufficient time for coders to become familiar with the data and conduct coding and the use of multiple coders. Thick description, including describing participants’ experiences and behaviors in the context of their lives, was utilized to ensure transferability. Dependability and confirmability are established through rigorous data collection protocols and record keeping.

## Results

**Fig. 1:**
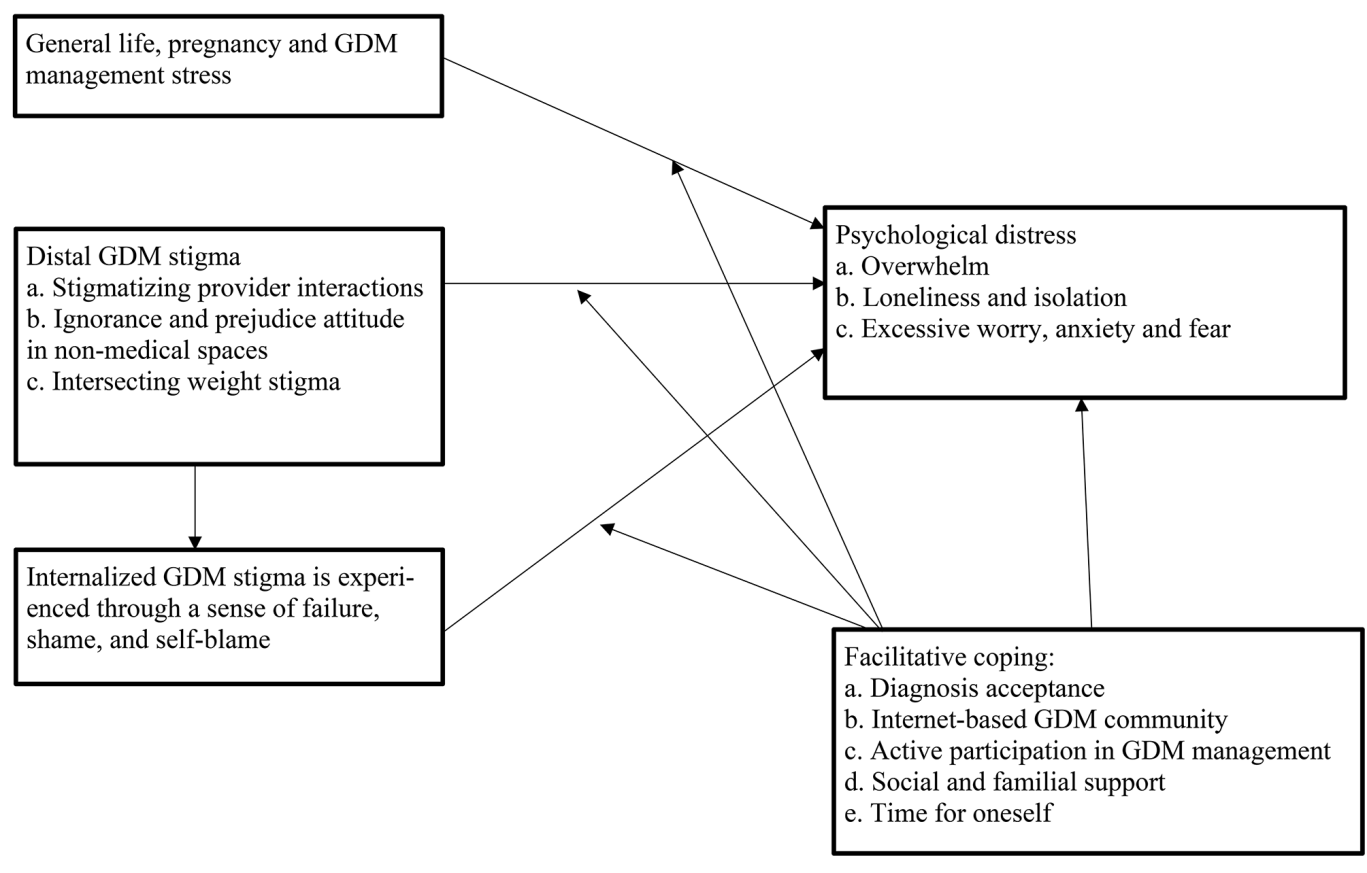
Framework on stigma, distress, and coping based on emerged themes

Analysis revealed four main themes, including (1) experience of distal GDM stigma, (2) internalized GDM stigma, (3) psychological distress, and (4) facilitative coping. Figure 1 presents a theoretical model that includes subthemes and illustrates the relationships among themes. As general life, pregnancy, and GDM management stress were not centered on GDM stigma, we included it in Fig. 1 as an influential factor to psychological distress without presenting it as a distinct theme in the Results. As illustrated, experience of distal GDM stigma contributes to internalized GDM stigma, and both forms of stigma lead to psychological distress, while facilitative coping may moderate the relationship between stigma and stress to distress. Below we describe each theme and their subthemes.

### Theme 1: distal GDM stigma is experienced in provider interactions and in non-medical spaces and often intersects with weight stigma

Most participants’ described experiences of stigma related to their GDM diagnosis coming from external sources (stigma placed on individuals due to the diagnosis). Three subthemes involved sources of distal GDM stigma including from (a) provider interactions, (b) non-medical spaces, and (c) intersecting stigma with weight.

#### Subtheme a: stigmatizing provider interactions

Participants shared experiences of stigmatizing interactions with their medical providers (OB providers, nurses, medical specialists). Some participants were aware of GDM risk due to a family history of Type 2 diabetes or high body mass index (BMI). For some participants discussing risks was perceived as educational, yet for others it could also be experienced as stigmatizing when not handled in a sensitive manner. For instance, Rachel (33 years old, White, 1st pregnancy, postpartum at study enrollment), shared that being constantly reminded that she belongs to a “high-risk group” prior to diagnosis was unhelpful:

*There*’*s definitely stigma in the medical industry, and they*’*ll say* “*it*’*s not your fault” all day, every day, but before I received that diagnosis, all I ever heard was* “*well you*’*re probably going to get gestational diabetes just understand that this might happen to you since your BMI is so high.” And just constantly in every single appointment. I love my OB; I*’*ve been seeing that doctor since I was 16, but still every time it was* “*well just know this could happen.”*

Participants shared that they also encountered stigma when diagnosed. Audrey (32 years old, White, 2nd pregnancy, 33 weeks gestational) shared that she was angry since “the nurse used a lot of ‘you failed’ language.” She noted:

*When they called with the results of the—that the one-hour number was too high and I needed to do the three-hour, her—the sentence she used was literally, “You failed your one-hour glucose test, so now you have to do the three-hour.“ I was like, “Well, by how much? How high was it?“ She was like, “Oh, you failed it good.“ I was like, well, screw you. Then, I did the three-hour, and obviously did not pass that, and she called and was like, “You failed the three-hour glucose test. Your numbers were—all four of your numbers were too high,“ and I was like, “Okay, but that’s not helpful.“ Pass, fail is a terrible way to talk about this. The sentence your nurse needs to use should be* “*The result of your three-hour glucose test are that you have GDM.” There is a stigma issue that comes from a lack of knowledge. I know that it*’*s not an uncommon complication of pregnancy, but the individual practitioners seem to not know enough about it to do anything besides applying a standard of care rather than individualized care. I am automatically getting dumped into this burdensome level of high-risk obstetric care.*

Participants also noted that the management approach to GDM felt top-down and fear-based. For instance, Elizabeth (34 years old, White, 2nd pregnancy, postpartum) noted that her nurse was “fear-mongering.” Rachel (33 years old, White, 1st pregnancy, postpartum) shared that “there was a lot of ‘if I eat this, I’m going to kill the baby’.” Katie (38 years old, White, 1st pregnancy, 35 weeks gestational) elaborated on her experience of “fear culture”:

*It*’*s very fear-based in my experience. It*’*s fear driven. They gave me a week and a half to get my sugars under control. I just was like*, “*That*’*s not enough time to figure out fasting, to figure out meals, to figure out how much you have to walk or exercise.” My fasting was out of control for two weeks before I got it back in control. It was always the fear of like*, “*Well, if you don*’*t get this together, we*’*re gonna give you to the endocrinologist, and the endocrinologist is gonna put you on metaformin or insulin.” They make it seem like being put on metaformin or insulin is a punishment, not necessarily a next step in treatment, which is another thing I had to learn from other women on an online support group, which is ridiculous. There*’*s a lot of fear culture, and when they use these tactics to scare the shit out of you, it really bothers me because I*’*m already doing the work.*

These interactions with providers have left participants feeling like they were being treated as a label, being blamed for their diagnosis, or not respected. Such interactions could impact patient-provider relationship, such as a lack of trust with providers, as well as patients’ subsequent experience with OB care.

#### Subtheme b: ignorance and prejudiced attitudes from non-medical spaces

Participants also shared experiences of stigma from non-medical spaces, such as friends, family, and co-workers. For instance, Charlotte (34 years old, White, 1st pregnancy, 31 weeks gestational) shared her encounters with ignorance and the burden to educate people:

*Another thing that makes it harder is that other people do not understand gestational diabetes. One of my husband*’*s co-workers made a stupid joke like*, “*Oh, you have to worry about your foot falling off.” I was like*, “*No, it*’*s something that only happens in pregnancy.” And he goes* “*Oh, so then you can eat whatever you want.” “No, I have to watch my carbs.” “Oh carbs, well you have to cut this, this, and this.” And then they all think they are experts and I*’*m like you don*’*t know, stop. But I also – I don*’*t want to go into explaining this to you. So I was trying to find ways to discuss that makes it short. That can be a little triggering, where it*’*s like* “*You are not listening to me, you don*’*t understand” and then also* “*I don*’*t really care enough to teach you because it*’*s not information that will be important to you, it*’*s only to mine.”*

As Charlotte shared, stigmatizing attitudes from non-medical spaces may be similar to stereotypes about Type 2 diabetes and also show up as uninvited advices. The lack of understanding and prejudiced attitudes about GDM can result in feelings of isolation for many, which we describe more fully in Theme 3.

#### Subtheme c: intersecting stigma with weight

GDM stigma experienced by participants often intersected with weight in both medical and non-medical spaces. Weight-based stigma can be experienced as feeling GDM is attributed to one’s weight. For instance, Rachel (33 years old, White, 1st pregnancy, postpartum) shared that she was referred to a specialist prior to her GDM diagnosis, who made comment about her weight:

*I was at 20 weeks, gone in for my anatomy scan, and they had actually referred me to the fetal specialist early and I hadn*’*t been diagnosed yet. I passed that 3-hour glucose tolerance test and was good for another 6–8 weeks without taking it, but they had referred me back to a fetal specialist and I ended up seeing one who was not great at all. I sat down and he goes* “*Oh so you are here because of your weight” and I was like* “*I don*’*t know why I*’*m here, I was told to follow up with you guys,” and he was like*, “*Oh well, I haven*’*t read your files yet.” And then he read it and he was like* “*Oh your baby*’*s tracking at 22 weeks, so he*’*s going to be overly large so you know you probably have gestational diabetes.” And I was like* “*Well I have passed the three hour test.” And he said*, “*So well that doesn*’*t mean anything, you have to take it again.” He was just so in my face about my weight, it was just constantly like well why didn*’*t you lose the weight? Why did you get pregnant now?*

Weight-based stigma also occurs in non-medical spaces, often discussed by participants in the context of explaining GDM to their friends and family. For instance, Kelly (32 years old, White, 1st pregnancy, 32 weeks gestational) shared:

*I definitely encounter stigma with a lot with people in my life. Even somebody the other day—it’s actually a good friend, but she’s like, “Yeah, this person has diabetes ‘cause they’re fat and whatever, and you’re just like—and lazy.“ I was like, “No. It’s not always like that.“ I get frustrated with that.*

The underlying assumption that weight causes the GDM condition can bring a sense of shame and self-blame for those who are overweight and frustration when one does not fit into this misconception.

### Theme 2. internalized GDM stigma is experienced through a sense of failure, shame, and self-blame

As a reaction to the distal stigma participants experienced, many described examples of internalized GDM stigma. One way that GDM stigma “gets under the skin” is through internalization. Participants shared internalized GDM stigma as a reaction to distal stigma, often experienced as “a failure,” shame/guilt, and self-blame. For instance, Rachel (33 years old, White, 1st pregnancy, postpartum) noted:

*I just felt really guilty knowing that this pregnancy was something we really wanted and worked hard for. So, knowing that my BMI is so high and I never bothered to lose all the weight and never bothered to get my lifestyle under control and change my diet before I got pregnant. I felt like a failure, like I had done this.*

Even for participants who understand that GDM is caused by hormones released by the placenta during pregnancy, the sense of guilt could still be present. Elizabeth (34 years old, White, 2nd pregnancy, postpartum) noted:

*I just felt this is just all my fault. I made a crappy placenta. I can*’*t even know how*’*s my baby. You know I just felt really guilty and really bad.*

Self-blame also occurs when participants are not completely adhering to their diet or exercise plan. This is often discussed in the context of “mom guilt” and a sense of failure and shame when pregnant individuals do not achieve an ideal lifestyle or glucose levels in a perfect manner. For instance, Emma (32 years old, White, 1st pregnancy, 27 weeks gestational) shared:

*I know and it’s really easy to blame yourself, because you know - I find that they want you on this gestational diabetes diet to eat like six times a day which is friggin crazy. And I think about all those times that I skipped my snack between lunch and dinner and I’m like* “*you did this, you should have eaten something”, but like also that’s just not - that’s not the truth, like I know it’s not my fault but it’s very easy to blame yourself as the mother.*

These internal experiences of GDM stigma, including a sense of failure, shame, and self-blame, respond to distal GDM-related stigma (described in Theme 1) where participants encounter environments and interactions that induce such feelings. These internalized stigma experiences can contribute to feelings of stress, and excessive worry and fear, which we describe in Theme 3.

### Theme 3. psychological distress is a common experience following GDM diagnosis and is associated with experiences of stigma

GDM diagnosis, followed by lifestyle adjustment, as well as stigmatizing experiences (both distal and internalized; e.g., feeling like a failure, experienced GDM management as “fear-driven”), had significant impacts on participants’ psychological health. In addition, some participants noted general life stress (e.g., other children in the household, house renovation, job change, etc.) also contribute to participants’ mental health. Psychological distress manifested in different ways including (a) stress and overwhelm, (b) anxiety, worry, and fear, and (c) loneliness and isolation.

#### Subtheme a. stress and overwhelm

Feelings of stress and being overwhelmed were often discussed in the context of GDM management, especially following initial diagnosis. For instance, Florence (36 years old, White, 4th pregnancy, 36 weeks gestational) shared:

*Being diagnosed, it was terrifying. You google everything. Google*’*s the worst. It tells you the worst-case scenarios. I*’*m anxious as it is, so hearing all that made it that much more serious for me. At the beginning, I was crying a lot because it just feels – I*’*ve never dieted. It was hard. It*’*s a whole life change. You have to plan your whole life about it. It was overwhelming beginning the diabetes diet and just having so many restrictions on everything. You do pity yourself. It*’*s stressful, especially relearning everything.*

Participants also noted that experiences of stress and overwhelm may feel “subclinical” but significant to one’s well-being, and traditional ways of thinking about mental health may fail to capture one’s experience. For instance, Mako (31 years old, Asian, 1st pregnancy, 31 weeks gestational) noted:

*My feeling of overwhelm may not be exactly as classic depression or anxiety. In a very short amount of time and majority of my pregnancy, my life has gone through a lot of changes. I think it*’*s just that my—with everything going on, it*’*s just—there*’*s a lot to think about…I think I would describe my well-being as being overwhelmed a lot of the time. Yeah. You know, one thing I read in a post this morning was someone got diagnosed and she said I feel paralyzed. Paralysis because of not knowing what to do or how to – what it – or how to just make sense of it. I think it*’*s those types of things that – it*’*s not classic symptoms or characteristics of what we consider mental health concerns, but they are kind of underlying.*

The diagnosis of GDM often calls for various lifestyle adjustments (changes of routine, meal planning, etc.) along with other changes and anticipation of changes that take place during the period of pregnancy. The experiences of stress and overwhelm may be natural responses to new demands in GDM management yet also deserve clinical attention and potentially mental health prevention intervention.

#### Subtheme b. excessive worry, anxiety and fear

Participants overwhelmingly shared feelings of anxiety, worry, and fear about baby’s health, pregnancy, labor, and delivery in the context of GDM. For instance, Olivia (30 years old, White, 4th pregnancy, 32 weeks gestational) shared:

*My biggest anxiety is just not dying at any given point. I*’*m like well is all these things are going wrong, like my heart could stop at any moment, stroke, embolism, even though my blood pressure and sugars have been totally fine – I do these both four times a day and I*’*ve never had anything actually over 120. But still I just have all things I feel in any moment anything could do wrong and I could die.*

Some participants also shared their anxiety shaped by past experiences of pregnancy complications by oneself (e.g., PCOS, previous miscarriage) or a close family member (e.g., a participant disclosed feeling afraid of delivery due to GDM and grandma lost her life in childbirth from hemorrhage). For instance, Audrey (32 years old, White, 2nd pregnancy, 33 weeks gestational) shared:

*GDM has definitely made me more anxious. I don*’*t have any background diagnosis or anything like that, but definitely the anxiety has gone up because of GDM. It*’*s like, is the baby doing okay? Am I gonna need a c-section? All these sort of questions are now more – they stick around in my brain more. I think that in general, it was – as a background history, I*’*ve also had two miscarriage and so – and the first one was the first time I got pregnant, so I*’*ve never had an easy pregnancy because my first ended in miscarriage, and so I*’*ve always had in the back of my head, things do go wrong with pregnancies. I can*’*t point to any specific thing, but I feel like there*’*s this just overall increase in the things I*’*m thinking about the things that ruminate and stick around and prevent me from focusing on the task at hand or shutting my brain off when I*’*m gonna try to go to sleep.*

As shown above, excessive worry and anxiety may manifest in rumination, catastrophizing, and poor sleep quality. They may also be reinforced by stigma and interactions that might emphasize adverse outcomes of GDM.

#### Subtheme c. loneliness and isolation

Participants discussed experiences of loneliness and social isolation as a form of psychological distress in the context of GDM management, lacking a community of people with GDM, and stigma preventing them from having open discussions with others about their experience. For instance, Emma (32 years old, White, 1st pregnancy, 27 weeks gestational) shared that “I felt pretty shitty. And I’m embarrassed to tell people because of stigma.” Elizabeth (34 years old, White, 2nd pregnancy, postpartum) elaborated:

*Gestational diabetes felt like really isolating to me. I didn*’*t know anybody who had it. I mean only one of my friends who*’*s ever got it so if I didn*’*t search out groups online I would have had no one to talk to.*

Even for those with good social support, loneliness and isolation may still be a common experience due to the uniqueness of each pregnancy. For instance, Mako shared her insight:

*At the end of the day, pregnancy itself can be a very isolating experience because not everyone around you is going through the same thing as you are. You might know other pregnant women. They might be within a few days of your due date, but they have such different experiences. Even a best friend, a spouse, a sibling, there*’*s only so much they can tell you because they don*’*t know what is actually going on.*

As illustrated, GDM stigma and the lack of community or connections with other individuals with GDM can be detrimental to patients’ well-being. It also demonstrates participants’ desire in sharing and discussing their experiences and a need for social connections.

### Theme 4. participants utilized a multitude of facilitative strategies to cope with experiences of stigma and psychological distress

Despite the common experiences of distal and internalized stigma and psychological distress, most participants developed a variety of strategies to overcome these experiences. These strategies comprised of (a) diagnosis acceptance, (b) internet-based GDM community, (c) active participation in GDM management, (d) social and familial support, and (e) time for oneself.

#### Subtheme a. diagnosis acceptance

Participants shared that confronting the diagnosis of GDM was emotionally difficult, and acceptance helped them to overcome psychological barriers and focus on effective GDM management:

*Being diagnosed was very frustrating. I*’*m a particularly healthy person. We eat very well. It was a bit shocking. It was also shocking to find out that the way they tested was disgusting to me. The syrup that they gave me – I was shocked that there was 50 g of sugar. I was just like*, “*How else was my system support to react except to go completely crazy, especially for someone who doesn*’*t eat a lot of sugar.” I had a great deal of cognitive dissonance. Once I get past that cognitive dissonance of this is bullshit and the way you test this is bullshit, it was just, okay, let*’*s get into go mode and make sure that I*’*m healthy and she*’*s healthy because it*’*s more about her than anything else.*

#### Subtheme b. internet-based GDM community

Participants overwhelmingly shared that reading/knowing other people’s experience with GDM and receiving online support from internet-based GDM communities (e.g., Facebook groups, Reddit forum) were crucial for their GDM management and to combat stigma. For instance, Mako (31 years old, Asian, 1st pregnancy, 31 weeks gestational) shared:

*I found that the Reddit sub was really helpful to at least get to feel comfortable with diagnosis because there were so many people on there, like I just got diagnosed, I don*’*t know what to do. And then people would say at first we were really upset, but we found there have been benefits to it. Some people end up finding that they can build more stamina and build more endurance, and it helps them for labor and then post-partum recovery. I really found that just in general in pregnancy, being able to commiserate with people who are in the similar—who are in a similar boat as me has helped a lot.*

Katie (38 years old, White, 1st pregnancy, 35 weeks gestational) noted that the online group acted as a support group and helped normalize her experience:

*The Reddit group, I call them my support group. There*’*s lots of threads in there that will start out*, “*Am I crazy?” Yeah. There*’*s a lot of threads where it*’*s like*, “*Am I crazy?” dot, dot, dot. It*’*s nice to hear other women be like*, “*You*’*re not fuckin*’ *crazy.” That puts a lot of ease.*

Emma also shared how connecting with others with GDM online helped her to reduce isolation and stigma:

*I have learned like 90% of what I know about gestational diabetes and like life hacks around gestational diabetes through the Reddit forums, because nobody knows gestational diabetes like somebody who*’*s living with it. Being able to hear other people*’*s experiences and also again just knowing – if I could see a healthy person that looks like me – I don*’*t know anyone with gestational diabetes. It has such a stigma. If I could see that, like I*’*m not the only one, I think it might have made me less – I felt pretty shitty for a couple weeks there. And I*’*m embarrassed to tell people. It*’*s embarrassing because of the stigma, so I think it would make me feel less stigmatized if I know there are people who look like me. (Emma, 32 years old, White, 1st pregnancy, 27 weeks gestational)*

This coping mechanism directly responds to the experiences of isolation and loneliness and also serves as a space to normalize one’s experiences of stigma and provide social support. Internet-based GDM community may help individuals express their voices in a relatively safe and de-stigmatized environment.

#### Subtheme c. active participation in GDM management

Participants discussed active participation in GDM management, motivated by health of the baby, helped them to regulate their glucose level, learn about their own health, and gain a sense of self-efficacy. Many shared their experience of GDM management in the context of learning strategies from the internet. For instance, Laura (35 years old, White, 4th pregnancy, 19 weeks gestational) noted:

*But I feel like most of what I’ve learned about GD, I’ve researched myself either through, obviously, Google or a couple of different Facebook groups and just reading other moms*’ *experiences. That has definitely been helpful.*

Florence (36 years old, White, 4th pregnancy, 36 weeks gestational) shared how she started learning about and managing GDM prior to her official diagnosis made by her medical care team:

*I’ve never even met anybody that had had it. Whenever I did the one-hour glucose, I just never really thought twice about it. This time, I failed it. When I failed it, I had a feeling. I joined the Facebook groups, tried to start learning about it. Then I got my three-hour results, and I did just failed it terribly. The next day, I went out and got my meter. Started learning from what my group had said. Started implementing that in my daily life. It took a week or two for my doctor’s office to get a hold of me. I was glad I found that Facebook group to try to get my numbers under control. I’ve been diet-controlled this whole time. I’ve only had a few high numbers.*

As Florence shared, her quick actions, active participation, and engagement in GDM management helped her to gain control of her glucose level. As glucose dysregulation can be a source of stress and anxiety for individuals, active self-management could also potentially help with psychological health.

#### Subtheme d. Social and familial support acts a buffer to distal and internalized GDM stigma

Participants identified support from family and friends as an important source of coping. For instance, Sachiko (24 years old, Asian, 1st pregnancy, 29 weeks gestational) noted, “I do think having the support system for me is helpful, just being able to talk to my husband or spend time with my parents and tell my closer friends.” Similarly, Rachel (33 years old, White, 1st pregnancy, postpartum) shared support from her husband when she experiences distress:

*My husband – he was really good. I*’*m becoming emotional. Yeah, it was really hard. It was just constantly stressful and did I cause this and maybe a bit of depressive feelings and thoughts about, you know, causing this and this being my fault. And he really did a good job taking a lot of that mental load and saying this isn*’*t your fault, this is what we are going to do. Making sure, giving me ideas for different kinds of foods and he joined a reddit page like a for supporters to like figure out ways he could—what kind of food he could put in front of me, and what he can say and kind of distracting me with hiking and our favorite activities and things like that.*

#### Subtheme e. making time for oneself

Participants shared having time for oneself, particularly engaging in sensory activities such as massages and baths, helped them to access their bodily sensations, improve mood, and reduce stress. For instance, Kelly (32 years old, White, 1st pregnancy, 32 weeks gestational) shared:

*I actually just got a prenatal massage last week, and it was amazing, so I booked another one for—I think it’s in two weeks. I was very excited about that. I love showers, sitting in the shower. I sit down in the shower sometimes when I’m stressed, and I just love it. It relaxes me.*

Susan (30 years old, White, 2nd pregnancy, 27 weeks gestational) noted similar activities (spa), as well as taking the freedom to have time for herself and “doing what she wants to do”:

*We went to a whole spa, and we stayed there. I had eight hours of treatments done. I needed it. It was amazing. It’s really been mostly just listening to my body and taking care of it that way. If I need a nap, I’m gonna take a nap. If I’m at work in my office, I’m just gonna shut the door and put my head down on the desk. Doing the things that I want to do. Even just watching TV and catching up on something or reading a book, which I’ve never done, or listening to a podcast instead of doing the dishes. My husband has been incredibly helpful with letting me just take time for myself ‘cause I think he also realizes we’re not gonna have any time for ourselves. I would say really just letting myself do things that I want to do.*

Changing the scene, such as taking a walk, was also identified as a relaxation activity by Emma (32 years old, White, 1st pregnancy, 27 weeks gestational):

*If I’m really upset I’ll just take the dog on a walk, put a podcast in or put some music on or call my husband. Being outside and away from all of the stimulants inside and just being able to enjoy being with my dog in nature feels so - I don’t know how to describe it, it just feels so much better and I never think about diabetes, the baby’s well-being, like I never think about it, I never have a negative thought when I’m on a walk.*

Rediscovering and engaging in one’s hobbies (e.g., reading, knitting) also helped participants cope with difficulties. For instance, Rachel (33 years old, White, 1st pregnancy, postpartum) shared:

*I picked up reading again. I haven’t had a lot of time to read for fun since college because [work] takes over my life and every one of my books is [work related], just so interesting. Taking up reading helped me to like get back into—and not just young adult novels, I read a lot of young adult novels mostly to like connect with my students—but picking up like adult novels and escaping somewhere that’s maybe not meant for a teenager, you know, that helps too.*

This coping strategy responds to the experiences of stress and overwhelm, and may also potentially help to re-connect with oneself and enhance agency.

## Discussion

The current study explored experiences of GDM stigma and stress and how such experiences impact psychological health among these individuals, as well as facilitative coping to combat them. Using thematic analysis, findings demonstrated (a) the relevance of GDM stigma among people diagnosed with GDM and (b) the need to conceptualize GDM stigma as a powerful source of impact on maternal health while individuals undergo GDM treatment. Study findings complement a recent review synthesizing evidence related to GDM stigma from varied studies [26], while investigating this question from a stigma-focused lens and with specific attention to facilitative coping strategies. With the increasing prevalence of GDM in the United States and globally [2, 32, 33], findings of this study provide insight into the psychosocial challenges that people with GDM face as well as strategies to address GDM stigma and stress and ultimately promote the well-being of parents and infants.

Research on stigma outside of pregnancy and with people with Type 1 or 2 diabetes has been growing recently [22–25]. Results from our study suggest that there are similarities between the two (e.g., experience of blame and judgment from others, internalized stigma experienced as shame, guilt, and diabetes being a personal failure). Findings also reveal some potential differences and unique aspects of stigma for individuals diagnosed with GDM. These may include people’s experience with their OB providers and a sense of failure in responsibility not only for oneself but more importantly their child (e.g., “blame yourself as the mother”). The short-term nature of pregnancy and GDM also appears to shape the experience of GDM stigma, as individuals with GDM may be less likely to endorse a patient identity for diabetes treatment (as compared to people with Type I or II diabetes), knowing that their diabetes status will likely change following birth.

A potential source of distal GDM stigma, as shown in the Results, stems from interactions with healthcare providers. Factors that influence the approach of healthcare providers appear to include historically how GDM has been managed in the OB medical system, such as labeling certain groups of patients as “high risk,” a cookie-cutter (i.e., numbers-focused) approach, fear-based management tactics, and negative language used in GDM discussion (e.g., “You failed your glucose tolerance test”). This resulted in participants feeling that they were not being treated as an individual patient but rather a category, seeking information outside of the healthcare system on GDM management (e.g., online spaces, sometimes perhaps also due to feeling a lack of information from their providers), and reinforced internalized stigma. Of note, these practices are not in alignment with the current body of literature on behavioral health promotion (e.g., how to best motivate patients to initiate desired behaviors), which emphasizes providing trust and autonomy, fostering self-efficacy, and enhancing knowledge, skills, and problem-solving abilities [34, 35].

As shown, weight stigma from healthcare professionals was also experienced by participants, as well as from other non-medical spaces. Outside of the GDM context, research on maternity care has shown that individuals with higher BMI reported more negative experience of care during pregnancy and OB providers perceived them as having poorer self-management behaviors and less positive attitudes towards caring for individuals with higher BMI [36]. Interestingly, a recent study with 358 women found that experience of weight stigma increased the odds of GDM, with a stronger association than BMI [37]. Although the mechanism was unclear [37], attending to weight stigma and the intersectionality of weight and GDM stigma represents an important area of research and clinical practice.

Experience of distal stigma may reinforce the internalized belief that GDM is a personal failure and the emotional response including a sense of shame and self-blame. Cumulatively, both forms of stigma, as well as GDM management-related stress, can affect the mental health of individuals with GDM. Findings of the current study highlight the adverse psychological impact experienced as overwhelm in the context of GDM management, anxiety and fear related to birthing (often shadowed by GDM complication and one’s personal or one’s family history in pregnancy and birthing), and overall isolation and loneliness in their pregnancy experience with GDM. Some studies suggest that people with GDM are more likely to report depression, anxiety, and stress during pregnancy [38, 39], and a recent meta-analysis found that people with GDM also experienced a significantly increased risk of postpartum depression (Relative Risk = 1.59) [40]. As psychological distress can be detrimental to glucose management (i.e., studies have shown that those with higher levels of distress are more likely to report elevated glucose) [41, 42], it is essential to understand factors contributing to mental health—including stigma—to inform the development of effective intervention programs to promote psychological health in this group.

A unique contribution of this study was the identification of facilitative coping strategies. Research in other stigma-related fields, such as the minority stress theory [43], poses that coping is an essential element of theory. Findings from this study complement this perspective and highlight the importance of conceptualizing facilitative coping in GDM stigma research. Uncovered strategies represent coping in psychological, social/familial, and medical spheres. Medical professionals and intervention programs that work with people with GDM may achieve greater outcomes by understanding and promoting these factors. These strategies may include engaging individuals and their support system, enhancing self-efficacy through GDM self-management, and attending to their psychological health. Findings on participants’ use of internet-based communities also suggest the potential utility of internet-based interventions to reach and engage people with GDM following their diagnosis to reduce stigma and isolation, access social support, and gain knowledge and skills related to GDM management.

### Study Strengths and Limitations

Study findings should be interpreted in the context of its strengths and limitations. Several strengths of the study are worth noting, including (a) a stigma-focused approach to understand the experiences of GDM-related stigma and build a conceptual framework, (b) use of qualitative method to emphasize and capture participants’ voices, (c) illustration of the psychological impact of GDM-related stigma and stress, and (d) inquiry on facilitative coping strategies that can help guide future interventions. The study also has several limitations. First, as a qualitative research with a small sample—where all participants identified as cisgender female and the majority were White and with a full-time job—findings may not be generalizable, especially among gender diverse people, people of color, and low-income individuals. Second, as this research was conducted during the COVID-19 pandemic (recruitment started May 2022), participants’ experience may be influenced by the pandemic. For instance, reports on isolation and utilization of internet-based groups may be amplified by pandemic-related factors (e.g., clinic’s policy on restricting family members during OB visit may reinforce experience of isolation). Quality of care may also be impacted due to the pandemic. Third, as our study required discussion with people about GDM, enrolled participants may represent a pool of individuals who were open about and comfortable discussing their experience with a research team and were self-motivated for GDM management.

### Implications for Future Research and Clinical Practice

Findings have implications for future research and clinical practice with individuals with GDM. First, there is a need for research to test hypotheses based on findings from the study to assess the strength of these relationships (e.g., the association of stigma and mental health, the moderating role of positive coping on the relationship between stigma and mental health). Theory-guided quantitative research will be suitable to understand the generalizability of findings and further understand mechanisms. Second, future research may be particularly interested in the intersection of weight and GDM stigma and further understand how weight stigma may shape people’s experiences of GDM diagnosis and management and their mental and behavioral health. Third, more research is needed regarding providers’ GDM stigma (as well as weight stigma) and how it may affect their interactions with patients and subsequent patient engagement. As findings from the current study suggest interactions with providers as an important source of GDM stigma, it is critical to identify effective strategies to address provider stigma and improve quality of care. This may include education and training to help providers recognize stigma, including implicit forms of stigma, its potential impact on their interactions with patients, and best practices on communication and facilitating GDM management. Fourth, future research on GDM stigma experiences with diverse populations are needed, such as pregnant or postpartum people of color, pregnant or postpartum sexual minority people, and pregnant or postpartum gender-diverse individuals. These groups have been identified as experiencing more vulnerability to GDM [44–46] and/or diabetes, as well as other types of minority stress due to social identities [47, 48].

With regard to clinical practice, psychological/behavioral health interventions that can address GDM stigma, promote coping, and enhance mental health are highly needed. An empowerment, skills-focused approach, coupled with internet-based, individual and/or group-based delivery may be fruitful on this front. Programs to reduce provider interaction stigma and provide medical professionals tools to recognize and discuss GDM stigma and empower their patients for effective GDM management are also crucial. Finally, at a higher level, sociocultural changes and policy support are needed to increase societal awareness of GDM and reduce stigma in non-medical spaces.

## Conclusions

Gestational diabetes mellitus is a rising epidemic in the United States and globally, and there is an increasing need to understand GDM-related stigma and its relevance to mental health and GDM management. Utilizing qualitative research, the current study builds a theoretical framework and illustrates the experiences of distal and internalized GDM stigma among individuals diagnosed with GDM, the adverse impact of stigma on psychological health, as well as coping strategies that could potentially alleviate the negative consequences of GDM-stigma and promote mental and behavioral health. The study findings call for further research in measuring and understanding GDM stigma, quantifying its potential impact, and development and evaluation of interventions that may reduce stigma at multiple levels (e.g., patient and provider).

**Table 1:** Sample demographic characteristics

Sociodemographic characteristics	*N* (%)
Pregnancy status	
Currently pregnant	11 (78.6%)
< 1 year postpartum	3 (21.4%)
Education	
Some college	1 (7.1%)
Bachelors or Associates	5 (35.7%)
More than college	8 (57.1%)
Employment	
Unemployed / Not working	4 (28.6%)
Working Full-time	9 (64.3%
Working Part-time	0 (0.0%)
Missing / Not answered	1 (7.1%)
Race/Ethnicity	
Non-Hispanic, White	12 (85.7%)
Non-Hispanic, Asian	2 (14.3%)
Geographic Region	
Midwest	3 (21.4%)
New England	2 (14.3%)
South	6 (42.9%)
West	3 (21.4%)
Household income	
Less than $10,000	0 (0.0%)
$10,000 to $19,999	0 (0.0%)
$20,000 to $34,499	0 (0.0%)
$35,000 to $49,999	2 (14.3%)
$50,000 to $74,999	1 (7.1%)
$75,000 to $99,999	0 (0.0%)
$100,000 or more	11 (78.6%)
	*M* (*SD*)
Age	32.29 (3.31)
Gestational age (if currently pregnant)	31.31 (5.09)

## Data Availability

The data collected for the study are not publicly available due to interview data being identifiable. Codebook and other related data materials (e.g., de-identified transcripts) will be available from the corresponding author on reasonable request.
